# *Rhodnius prolixus* Hemolymph Immuno-Physiology: Deciphering the Systemic Immune Response Triggered by *Trypanosoma cruzi* Establishment in the Vector Using Quantitative Proteomics

**DOI:** 10.3390/cells11091449

**Published:** 2022-04-25

**Authors:** Radouane Ouali, Larissa Rezende Vieira, Didier Salmon, Sabrina Bousbata

**Affiliations:** 1 Proteomic Plateform, Laboratory of Microbiology, Department of Molecular Biology, Université Libre de Bruxelles, 6041 Gosselies, Belgium; 2 Institute of Medical Biochemistry Leopoldo de Meis, Centro de Ciências e da Saúde, Federal University of Rio de Janeiro, Rio de Janeiro 21941-902, Brazil; larissanat@hotmail.com (L.R.V.); salmon@bioqmed.ufrj.br (D.S.)

**Keywords:** Chagas disease, proteins expression, triatomines, antiparasitic response, insect immunity

## Abstract

Understanding the development of *Trypanosoma cruzi* within the triatomine vector at the molecular level should provide novel targets for interrupting parasitic life cycle and affect vectorial competence. The aim of the current study is to provide new insights into triatomines immunology through the characterization of the hemolymph proteome of *Rhodnius prolixus*, a major Chagas disease vector, in order to gain an overview of its immune physiology. Surprisingly, proteomics investigation of the immunomodulation of *T. cruzi*-infected blood reveals that the parasite triggers an early systemic response in the hemolymph. The analysis of the expression profiles of hemolymph proteins from 6 h to 24 h allowed the identification of a broad range of immune proteins expressed already in the early hours post-blood-feeding regardless of the presence of the parasite, ready to mount a rapid response exemplified by the significant phenol oxidase activation. Nevertheless, we have also observed a remarkable induction of the immune response triggered by an rpPGRP-LC and the overexpression of defensins 6 h post-*T. cruzi* infection. Moreover, we have identified novel proteins with immune properties such as the putative c1q-like protein and the immunoglobulin I-set domain-containing protein, which have never been described in triatomines and could play a role in *T. cruzi* recognition. Twelve proteins with unknown function are modulated by the presence of *T. cruzi* in the hemolymph. Determining the function of these parasite-induced proteins represents an exciting challenge for increasing our knowledge about the diversity of the immune response from the universal one studied in holometabolous insects. This will provide us with clear answers for misunderstood mechanisms in host–parasite interaction, leading to the development of new generation strategies to control vector populations and pathogen transmission.

## 1. Introduction

Vector-borne diseases (VBDs) account for more than 17% of all infectious diseases, causing more than 700,000 deaths annually [1]. They can be caused by either parasites, bacteria, or viruses. These diseases are mainly transmitted to humans and other mammalians by hematophagous arthropods, such as flies, ticks, and bugs. These arthropods acquire pathogens when they ingest a blood meal from an infected host and eventually transmit them to the next host through vectorial competence [2]. Subsequently, transmission represents a vulnerable and attractive point of control. In addition, vector control remains the most effective method for preventing VBDs transmission in the absence of a safe and effective preventive alternative [2]. Insect vectors have a powerful immune system that has evolved to respond to these different pathogenic agents and the immune challenges they may face during their life cycle, especially when feeding on different hosts, as well as to control symbiotic bacteria [3]. Triatomines constitute a large group of vectors of the kinetoplastid protozoa *Trypanosoma cruzi*, the causative agent of Chagas disease [4,5]. We estimate about 6–7 million people infected with *T. cruzi* and 75 million people are at risk of infection resulting in approximately 10,000 deaths per year worldwide [1]. Triatomines acquire parasites when they feed on infected mammals. The ingested parasites develop exclusively within the digestive tract through the transition by different stages, before achieving the infective form, which is excreted with the feces and infects the vertebrate host [6]. Unlike *T. cruzi*, *T. rangeli*, a closely related non-pathogenic parasite to humans, migrates to the hemolymph through the intestinal epithelium, where it multiplies and then invades the salivary glands wherein it differentiates into infective metacyclic trypomastigotes from which it is transmitted by regurgitation (6–8). This difference between the two congeneric species is principally due to the ability of *T. rangeli* to inhibit the vector immune response in the hemolymph [7,8,9,10]. As all invertebrates, triatomines immune response is characterized by two types of innate responses: humoral response, which is related to antimicrobial peptides (AMPs), lectins, nitric oxide (NO) and the prophenoloxidase (PPO) cascade, and the cellular response which includes phagocytosis, hemocytes aggregation, and encapsulation of pathogens [11]. Moreover, triatomines are capable of specifically recognize and selectively eliminate infective agents [11,12]. Immune factors are secreted by the hemocytes, which circulate in the hemolymph: the insect’s circulatory fluid contained in the hemocoel, a counterpart of vertebrate blood [13]. The additional immune factors are secreted by the fat body, a mesodermal tissue distributed throughout the hemocoel [3]. The hemolymph constitutes the center of most physiological processes. Indeed, it delivers nutrients and hormones to tissues, removes waste products, maintains homeostasis, and more importantly coordinates defense mechanisms [13,14].

Many of the abundant hemolymph proteins have been identified and their functions elucidated [14]. Amongst them are storage proteins or hexamerins which are the source of amino acids, lipophorins involved in lipid transport and catabolism, vitellogenins which play an important role in embryogenesis, and proteins involved in the innate immune response [14]. Hence, important efforts have been deployed to decipher the molecular mechanisms controlling insects’ immunity [15] which have largely been focused on holometabolous insects such as *Drosophila* [16], *Aedes* [17], and *Anopheles* [18]. Because of the importance of hemolymph for insects in surviving against invading pathogens and its importance as an indicator of global physiological changes, investigation of its proteome pattern is valuable and should lead to the discovery of new targets for pesticide research and development of new approaches to the management of vector-borne diseases. Since post-genomic approaches cannot be used for fluid tissues such as hemolymph, their analysis is restricted to proteomics. Indeed, several proteomics studies of fruit fly [19,20,21], silkworm [22,23], mosquitoes [24,25], and honeybee [26] hemolymph in different biological contexts (infection, nutrition, growth…) have been investigated.

This study aims to improve knowledge on triatomine hemolymph physiology and their immune response, by analyzing the effects of blood ingestion and digestion as well as the development of *T. cruzi* within the vector digestive system on the modulation of the hemolymph protein expression and secretion, using label-free quantitative proteomics. The investigation of the effect of these challenges on the hemolymph physiology should provide an overview of triatomines immunology as well as the characterization of the main actors of the immune response triggered by the protozoan at the systemic level. Particular attention will be paid to immune proteins, especially those modulated by the parasite to better understand the mechanisms that orchestrate the interaction with the vector and identify potential transmission blocking and vector control strategies.

## 2. Materials and Methods

### 2.1. Samples Preparation

*R. prolixus* insects were reared at a stable temperature of 28 °C and 60–80% humidity, under a photoperiod of 12 h light/12 h dark (Federal University of Rio de Janeiro, Rio de Janeiro, Brasil). Insects were fed with rabbit blood. Two groups of insects were used in this study, insects of the first group were fed through a latex artificial membrane feeding apparatus with normal heparinized rabbit blood (2.5 units/mL) and those of the second group were fed with *T. cruzi* (Dm28c clone) (Carabobo, Venezuela) infected rabbit blood (10^7^ epimastigotes/ml).

### 2.2. Hemolymph Preparation

Hemolymph was recovered from *R. prolixus* adult females (five insects/replicate) at 6 h and 24 h post-feeding. After disinfecting the cuticle with 70% ethanol, the third hind legs were cut off and the abdomen was gently pressed to induce the flow of the hemolymph that was collected into a microtube using micropipette. An equal volume of PBS (pH 7.4) was then added to the collected volume. The tubes were kept on ice during the process and then immediately stored at −80 °C until use.

### 2.3. Sample Preparation Prior to Liquid Chromatography Tandem Mass Spectrometry (LC-MS/MS)

The hemolymph was centrifuged at 5000× *g* for 5 min to discard hemocytes before protein digestion. For sample preparation we used the iST-BCT kit for bottom-up proteomic sample preparation according to the manufacturer’s instructions (PreOmics GmbH, Martinsried, Germany). In brief, 15 µg of hemolymph proteins were solubilized with lysis buffer and subsequently proteolyzed with trypsin. Tryptic peptides were washed, eluted from the IST column, and speedvac dried. After drying, the peptides were resuspended in solvent A (0.1% TFA in water/ACN (98:2, *v*/*v*)) and approximately 2 µg of each sample was injected for LC-MS/MS analysis on an Ultimate 3000 RSLC nanoLC (Thermo Scientific, Bremen, Germany) in-line connected to an LTQ-Orbitrap Elite (Thermo Fisher Scientific, Bremen, Germany) equipped with a pneu-Nimbus dual ion source (Phoenix S&T, Chester, PA, USA). Trapping was performed at 10 μL/min for 4 min in solvent A on a 20 mm trapping column (made in-house, 100 μm internal diameter (I.D.), 5 μm beads, C18 Reprosil-HD, Dr. Maisch, Germany) and the sample was loaded on a 200 cm long micro pillar array column (PharmaFluidics, Gent, Belgium) with C18-endcapped functionality mounted in the Ultimate 3000’s column oven at 50 °C. For proper ionization, a fused silica PicoTip emitter (10 µm inner diameter) (New Objective, Littleton, MA, USA) was connected to the µPAC™ outlet union and a grounded connection was provided to this union. Peptides were eluted by a non-linear increase from 1 to 55% MS solvent B (0.1% FA in water/ACN (2:8, *v*/*v*)) over 137 min, first at a flow rate of 750 nl/min, then at 300 nl/min, followed by a 13 min wash reaching 99% MS solvent B and re-equilibration with MS solvent A (0.1% FA in water).

The mass spectrometer was operated in data dependent, positive ionization mode, automatically switching between MS and MS/MS acquisition for the 20 most abundant peaks in each MS spectrum. The source voltage was 3.3 kV and the capillary temperature was 275 °C. In the LTQ-Orbitrap Elite, full scan MS spectra were acquired in the Orbitrap (*m*/*z* 300–2000, AGC target 3 × 10^6^ ions, maximum ion injection time 100 ms) with a resolution of 60,000 (at 400 *m*/*z*). The 20 most intense ions fulfilling predefined selection criteria (AGC target 5 × 10^3^ ions, maximum ion injection time 20 ms, spectrum data type: centroid, exclusion of unassigned and 1 positively charged precursors, and dynamic exclusion time 20 s) were then isolated in the linear ion trap and fragmented in the high-pressure cell of the ion trap. The CID collision energy was set to 35% and the polydimethylcyclosiloxane background ion at 445.120028 Da was used for internal calibration (lock mass).

### 2.4. Mass Spectrometric Data Analysis

Protein identification from the MS data was realized with the Andromeda peptide database search engine integrated into the computational proteomics platform MaxQuant (version 1.6.3.4, Max Planck Institute of Biochemistry, Germany) [27] with default search settings including a false discovery rate set at 1% on both the peptide and the protein level. Spectra were searched against *R. prolixus* proteins (UniProt Tax ID: 13249) in the UniProt/Swiss-Prot reference database (UniProt Proteome ID: UP000015103) and the decoy database. Andromeda search parameters for protein identification specified a first search mass tolerance of 20 ppm and a main search tolerance of 4.5 ppm for the parental peptide. Enzyme specificity was set to C-terminal to arginine and lysine, also allowing cleavage at arginine/lysine-proline bonds with a maximum of two missed-cleavages. Variable modifications were set to oxidation of methionine and acetylation of protein N-termini. A minimum of one unique peptide was required for identification. We allowed for matching between runs using a 1.5 min match time window and a 20 min alignment time window. Proteins were quantified by the MaxLFQ algorithm integrated in the MaxQuant software. A minimum ratio count of two unique or razor peptides was required for quantification.

Further data analysis was performed with the Perseus software (version 1.6.2.1, Max Planck Institute of Biochemistry, Planegg, Germany) after loading the protein groups file obtained previously by MaxQuant software. First, proteins identified by site and reverse database hits were removed and LFQ values were log2 transformed to achieve normal data distribution. Data from four biological replicates of each condition were grouped as two different conditions, and proteins with less than 2 valid values in at least one condition were removed. Then, missing values from the other condition were imputed with values from the lower part of the normal distribution representing the detection limit. Statistical significance of changes in abundance between sample groups was calculated by a two-tailed *t*-test, with *p*-values adjusted for multiple testing by a permutation-based FDR at 5%. Microsoft Excel was used to calculate ratios and fold-changes (FC) followed by log2 transformation.

Results are visualized by Volcano plots. A list of total identified proteins as well as differentially expressed proteins generated by Perseus software containing proteomic identification parameters (unique peptides, sequence coverage percentage, identification score LFQ and calculated FC) was then created.

### 2.5. Prediction of Protein Localization

Protein location was predicted by OutCyte prediction tool http://www.outcyte.com/analyse (accessed on 4 October 2021) [28]. The tool can predict whether the protein is intracellular, transmembrane, secreted by the signal peptide, or by unconventional protein secretion (UPS) based on physicochemical features directly generated from proteins amino acid sequences [28]. Proteins are predicted as transmembrane only if a transmembrane domain is present within the first 70 amino acids.

### 2.6. Functional Characterization and Protein Classification

UniProt ID numbers from the protein list generated by Perseus was searched against UniProtKB using Retrieve/ID mapping tool (https://www.uniprot.org/uploadlists) (accessed on 1 August 2021). This allows us to associate UniProt accession to the corresponding protein names, gene ontology categories and their IDs, molecular functions, protein families, subcellular locations, biological processes, signal peptides, MWs, post-translational modifications, and VectorBase IDs. Protein classification was then performed according to Gene Ontology (GO) hierarchy, using PANTHER (Protein ANalysis THrough Evolutionary Relationships) classification system (http://www.pantherdb.org/) (accessed on 1 August 2021) [28], and g:Profiler (https://biit.cs.ut.ee/gprofiler/gost) (accessed on 1 August 2021) [29]. Additionally, some proteins have been classified based on the presence of functional domains using InterPro (https://www.ebi.ac.uk/interpro/) and Pfam (http://pfam.xfam.org/) (accessed on 1 August 2021).

### 2.7. Phenoloxidase and Prophenoloxidase Activity

Phenoloxidase (PO) and prophenoloxidase activity in the hemolymph was determined by measuring the catalytic conversion of L-DOPA (3, 4-dihydroxy-L-phenylalanine) (Sigma, Overijse, Belgium) to dopachrome assayed at 490 nm. For the evaluation of the PO activity, 10 µg of hemolymph proteins were diluted in 100 µL of PBS. The mixture was added to 100 µL of L-DOPA at the concentration of 4 mg/mL. The mixture was incubated 20 min at 37 °C and then deposited in a 96-well microplate. The absorbance at 490 nm was measured continuously for 1 h, with a point measure every 5 min, using a microplate reader (SpectraMax i3, Molecular Devices, San Jose, CA, USA). The PPO activity was determined following the same protocol, led by the preincubation of hemolymph proteins with 5 µg of chymotrypsin (Sigma, Overijse, Belgium) before adding the L-DOPA substrate to activate PPO. All experiments were realized in triplicate. L-DOPA in PBS without hemolymph was used as negative control.

### 2.8. Western Blotting

15 μg of *R. prolixus* hemolymph proteins were labeled with cyanine CY5 (Serva, Heidelberg, Germany) for 30 min in the dark (four replicates for each condition were used). The reaction was stopped with 0.5 μg of lysine and labeled proteins were denatured in Laemmli buffer (4% SDS, 20% glycerol, 10% 2-mercaptoethanol, 0.004% bromphenol blue, and 0.125 M Tris HCl). Then, they were deposited on 10% SDS-PAGE gel. After separation, the proteins were transferred into a nitrocellulose membrane (Thermofisher, Merelbeke, Belgium) by electro-transfer for 75 min at 150 V at 4 °C, using the transfer buffer (0.25 M Tris, 200 mM Glycine, and 20% methanol). CY5 labeling was revealed at 700 nm by digital imaging with a CCD camera (Odyssey^®^ Fc, Bad Homburg, Germany) to quantify total protein signal (Figure S1). The membrane is then saturated overnight with TBS (20 mM Tris, 150 mM NaCl, pH 7.6) in the presence of 5% milk and 0.1% Tween20. The membrane was incubated for 3 h with the anti-defensin #ab225686 (Abcam, Cambridge, UK) in a solution of TBS, 2.5% milk, and 0.05% Tween20. The membrane was then washed 5 times with TBS, 2.5% milk, and 0.05% Tween20 to remove excess antibodies before introducing the secondary anti-rabbit antibody conjugated to horseradish peroxidase (Abcam, Cambridge, UK). The membrane was washed 3 times with a solution of TBS, 2.5% milk, 0.05% Tween20, and twice with TBS. The bound antibodies were detected by chemiluminescence. The light produced by the enzymatic reaction is detected by digital imaging with a CCD camera (Odyssey^®^ Fc, Bad Homburg, Germany). The relative expression of target protein was calculated by comparing the signal strength of the target protein to the total protein signal measured by CY5. The significance of the expression difference between the two conditions was calculated based on the difference of the means values between the two conditions using Student’s *t*-test.

## 3. Results

### 3.1. Overview of R. prolixus Hemolymph Proteome

**Figure 1:**
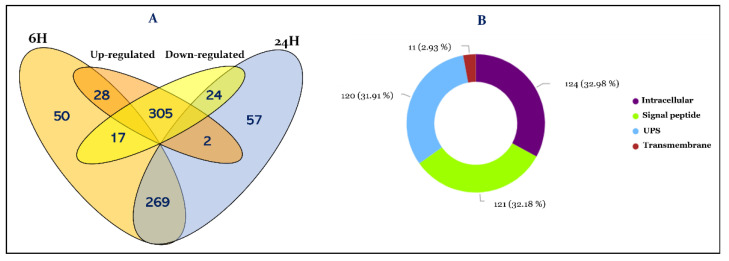
(**A**) Venn diagram showing the distribution of the identified proteins of the hemolymph at 6 h and 24 h post-infection. Intersections display protein expression specificity to each condition (Table S1); (**B**) Representation of the distribution of total hemolymph proteome between intracellular and extracellular proteins. Extracellular proteins are recognized using OutCyte prediction tool either by the presence of a predicted signal peptide using the SignalP algorithm, transmembrane or potential unconventional protein secretions (UPS) from intracellular proteins. Numbers in brackets indicate the percentage of proteins in each category.

In this study, we performed extensive proteomic analysis of the hemolymph of *R. prolixus* to gain functional insights into this tissue. Moreover, this analysis focusses on the immune modulation induced in response to *T. cruzi* challenge, highlighting the immune actors induced by the parasite at the systemic level. To achieve this goal the hemolymph from female adult bugs fed on normal blood and infected blood was obtained at 6 h and 24 h post-feeding. This fluid tissue was then analyzed by label-free shotgun comparative proteomics approach as described above. In total, we sequenced and identified 376 non-redundant proteins showing an important protein dynamic range spanning five orders of magnitude (Table S1). The investigation of the expression variation of these proteins allowed the identification of 269 constitutively expressed proteins from 6 h to 24 h post-feeding, and 107 time-dependent differentially expressed proteins, from which 50 are exclusively expressed at 6 h and 57 proteins at 24 h post-feeding (Table S1 and Figure 1A). Next, we focused on proteins regulated by the presence of *T. cruzi* compared to control, enabling us to identify 28 proteins at 6 h and 2 proteins at 24 h, whose expression is significantly induced in the hemolymph following the colonization of the digestive tract by the parasite. In contrast, the presence of *T. cruzi* negatively alters the expression level of 17 proteins at 6 h and 24 proteins at 24 h post-infection (Table S1 and Figure 1A).

The hemolymph ensures the exchange between the different tissues and consequently its proteome constitutes their secretome. Therefore, we investigated the localization of the identified proteins. Thus, among the identified proteins 121 are secreted through the classical endoplasmic reticulum-Golgi pathway with the guidance of a signal peptide (Table S1 and Figure 1B). However, proteins could also reach the hemolymph by following an unconventional secretory pathway (UPS). By using OutCyte [30], we have predicted 120 more proteins which are secreted by UPS. The remaining 124 proteins were predicted to be intracellular from which 11 were predicted as transmembrane proteins (Figure 1B).

### 3.2. Functional Annotation of Hemolymph Proteins

**Figure 2:**
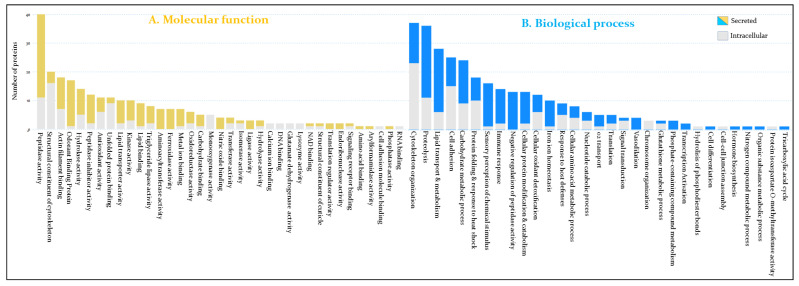
Functional annotation of *R. prolixus* hemolymph proteins. The proteins have been classified according to their molecular function (**A**) and biological process; (**B**) according to Gene Ontology. Exhaustive information about the identified proteins is provided in Table S1.

Hemolymph proteins were functionally characterized through GO term analysis using Panther and g:Profiler classification tools. The proteins were classified according to their GO molecular function and biological process (Figure 2). Hence, proteins with peptidase activity are the most represented functional category in the hemolymph with 40 proteins mainly involved in proteolysis and cellular protein catabolism (Figure 2 and Table S1). Among the identified peptidases, 29 are predicted to be secreted.

Proteins of cytoskeleton, such as actin, myosin, and tubulin are also among the most represented category with 38 proteins involved in actin filament organization and cytoskeleton assembly (Figure 2B and Table S1). On the other hand, 28 proteins amongst the most abundant proteins are involved in lipid transport and metabolism (Figure 2 and Table S1). Twenty-two proteins of this category are predicted to be secreted.

Twenty-five proteins of the extracellular matrix involved in cell adhesion and communication were identified, among which ten are secreted (Figure 2). Twenty-four proteins are involved in carbohydrate metabolism of which fifteen are secreted (Figure 2). In addition, eighteen proteins involved in protein folding, mainly related to heat shock cellular response have been identified, from which eight are predicted to be secreted (Figure 2).

**Figure 3:**
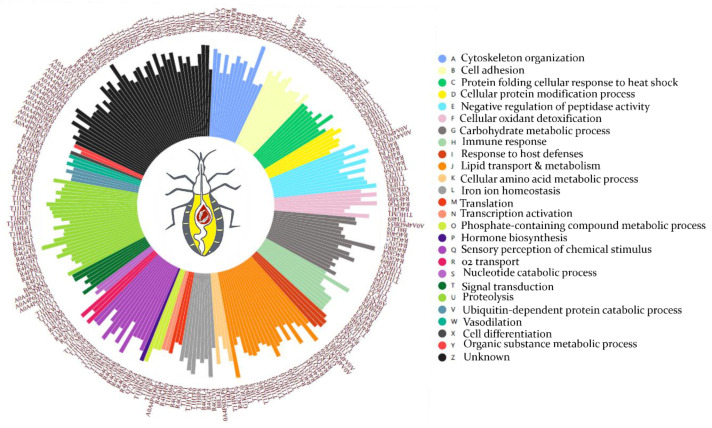
Circular histogram illustrating the distribution of *R. prolixus* secreted hemolymph proteins. The height of each bar is proportional to the LFQ intensity of expression of the corresponding protein, and each bar is related to the protein’s UniProt ID. Protein categories in the right panel are listed from the histogram clockwise.

Seventeen odorant binding proteins (OBPs) have been sequenced in this work and sixteen of them are secreted mainly through a signal peptide. Two OBP isoforms (T1H845 and R4G3B3) are amongst the most abundant proteins in the hemolymph proteome (Figure 3 and Table S1).

Sixteen proteins are directly related to the insect immune response according to GO biological process (Table S1), among which we have identified 2 lysozymes. T1I5M5 has a temporal expression profile repressed at 24 h post-feeding, while A9LN32 is expressed constitutively (Table S1). Moreover, their expression level is low compared to the other identified immune proteins (Figure 3). Seven proteins involved in pathogen recognition and pathogen-associated molecular patterns (PAMPs) have been identified and are represented by a protein containing an MD-2-related lipid-recognition (ML) domain (T1HU92) related to the recognition of pathogen-related molecules, a putative c1q domain protein (R4FJF3), GH16 domain-containing proteins (B8LJ39, T1HGN7, and T1I650), and peptidoglycan recognition receptors rpPGRP-LC/LAa and rpPGRP-LC/LAb (Table S1). Seven antimicrobial peptides (AMPs) comprising attacin-C domain-containing protein (T1I7V7), diptericins (D6BJP6 and E6Y430), prolixin (B8QEI8), and defensin domain-containing proteins (T1I7B0, R4G8B6, and R4FNJ9) were identified (Figure 3 and Table S1).

Thirteen peptidase inhibitors were identified, among which four serpins (T1IF83, T1I8D5, R4FLP4, and R4FJD2), three cystatins (R4FP01, T1I2F3, and A0A4P6D7E5), and a protein with four pacifastin inhibitor domains lcmii (R4G3U6), showing high level of expression (Figure 3). In addition, we have identified twelve cellular oxidant detoxifying proteins, including five Cu/Zn-superoxide dismutase (SOD) isoforms (R4FMI6, G1K083, T1HRT6, A0A4P6D9T0, and R4FPK6), two catalase domain-containing proteins (T1I0W4 and T1HV37), a glutathione peroxidase (T1I489), three glutathione S-transferase domain containing proteins (T1HVN9, R4G417, and T1HUM1), and two heme-binding proteins (Q8T5U0 and R4FPG0), these latter being amongst the most abundant proteins (Figure 3).

Interestingly, seven transglutaminases (TGc domain-containing proteins) with aminoacyl transferase activity, involved in post-translational modification have been identified (Table S1). Iron ion homeostasis process is also overrepresented by ten unique proteins, among which seven ferritins with ferroxidase activity and three transferrins. The expression level of these proteins is relatively high, in particular B8LJ43 (Figure 3). The next GO term is related to the melanization process of pathogens, which includes five POs (T1I7V8, A0A1B2G385, T1HW62, A0A1B2G381, and T1HW22), which together with lipid transporters and OBS constitute the most abundant proteins of the hemolymph.

Eight proteins involved in amino acids metabolism, five in oxygen transport, four in signal transduction, four in vasodilatation, two putative salivary lipocalins (T1HF25 and R4FN82), and two putative triabin-like lipocalins (R4G4J2 and T1H7Q9) have also been identified in this work. The other functional categories are underrepresented and belong to different insect physiological processes (Figure 2 and Table S1). Interestingly, 63 identified proteins are of unknown function due to the absence of GO and known functional domains, among which 45 are secreted (Table S1). Their expression profiles is steady at 6 h et 24 h (Figure 3 and Table S1).

### 3.3. Effect of T. cruzi on the Dynamic of Hemolymph Protein Expression

**Figure 4:**
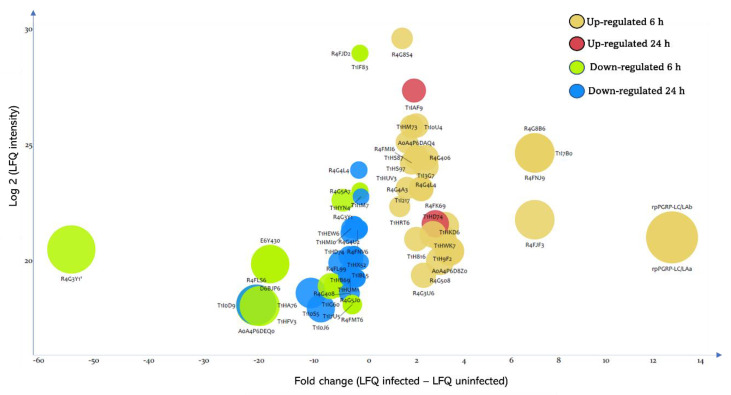
Bubble chart showing the differentially expressed proteins in *R. prolixus* hemolymph at 6 h and 24 h post-*T. cruzi* infection. Each bubble corresponds to a differentially expressed protein. x axis represents the fold change of protein expression, which is proportional to the bubbles size. y axis represents the log values of the intensity of protein expression.

The expression of 71 proteins is significantly modulated in the hemolymph following the ingestion of *T. cruzi*, among which, 45 proteins are regulated during the first hours following the establishment of the parasite infection and 26 proteins 24 h post-infection. Amid the differentially expressed proteins, 30 are significantly induced by *T. cruzi* and their expression increases up to 13-fold (Figure 4 and Table S1), and 41 are down-regulated, with a decrease in their expression level up to 54-fold (Figure 4 and Table S1).

Four proteases are up-regulated at 6 h post-infection, two of which are predicted to be secreted. These proteases are cysteine proteinase cathepsin L (T1HS87, T1HS97, and R4G406) and serine-type endopeptidase (T1H816) which expression is induced by two-fold in response to *T. cruzi* infection.

Among the two superoxide dismutases (SODs) induced by the parasite 6 h post-infection, only R4FMI6 is predicted to be secreted (Table S1). Interestingly, we noticed that *T. cruzi* strongly induces (seven-fold) the expression of the three defensins (T1I7B0, R4G8B6, and R4FNJ9) only at 6 h post-infection. In contrast, the expression of the two diptericins (E6Y430 and D6BJP6) is simultaneously strongly down-regulated by the parasite (eighteen-fold) solely at 6 h post-infection (Figure 4). The putative c1q domain protein (R4FJF3) shows a seven-fold expression level increase following infection and its expression profile follows that of defensins (Figure 4 and Table S1).

Among the highly induced proteins at 6 h post-infection were rpPGRP-LC/LAa and rpPGRP-LC/Lab showing expression increase by twelve-fold at 6 h and are completely repressed at 24 h (Figure 4 and Table S1).

The other up-regulated proteins at 6 h post-infection were pacifastin (R4G3U6), a putative chitinase (R4G8S4), an alpha-galactosidase (T1HM73), a ferritin (R4G4L4), three putative gamma-interferon-inducible lysosomal thiol reductases (T1HUV3, R4G4A3, and T1I217), a putative fasciclin (T1HD74), an OBP (T1I0U4), and five proteins with unknown functions (T1HWK7, T1I3G7, A0A4P6D8Z0, T1HKD6, and A0A4P6DAQ4) (Figure 4 and Table S1).

At 24 h post-infection, we identified that the putative hemolymph juvenile hormone binding protein (JHBP) (R4FK69) and the chemosensory protein (T1IAF9) are the only two significantly up-regulated proteins in the hemolymph in response to *T. cruzi* infection, while their expression was unmodulated by the infection at 6 h (Figure 4 and Table S1).

The expression of seventeen proteins is down-regulated in the hemolymph at 6 h following the ingestion of the parasite, among which four heat shock proteins 70 (HSP70) (T1I0D9, T1HA76, R4FLS6, and A0A4P6DEQ0) all being predicted intracellular. Their expression level decreases by twenty-fold upon infection (Figure 4 and Table S1). Furthermore, the presence of the parasite represses the expression of a transglutaminase (T1HFV3) by twenty-fold, which has been identified only in the hemolymph at 6 h (Table S1). The expression level of the predicted secreted peptidase S1 (T1I1M7) and two serpin domain-containing proteins (T1IF83 and R4FJD2) decreases by 1.6-fold by the presence of *T. cruzi* (Figure 4). The expression profile of the peptidase and their putative regulator are similar (Table S1). Surprisingly, the putative vitellogenin R4G3Y1 is strongly repressed by the parasite (Figure 4). Indeed, its expression decreases by 54-fold post-infection. The analysis of its expression profile indicates a prolonged down-regulation up to 24 h with a less apparent difference (five-fold).

Regarding the proteins affected negatively at 24 h by the parasite, we have identified four proteins involved in the metabolism of carbohydrates (R4G4U2, T1HB69, T1I0J6, and R4G5J0), a ferritin (R4G4L4), and a putative glutathione S-transferase (T1HUM1). Additionally, six unknown proteins are negatively modulated by *T. cruzi* ingestion, among which three are exclusively expressed at 24 h, such as T1I0S5, which expression decreases by ten-fold (Table S1).

## 4. Discussion

We investigated the effect of *T. cruzi* presence in the digestive tract of *R. prolixus* on the hemolymph proteome using quantitative label-free proteomics. Because *T. cruzi* colonization of the anterior midgut was previously shown to achieve peaks at 3 h post-infection and declines after 24 h post-infection [31], we compared hemolymph proteome from insect female adults at 6 h and 24 h post-blood feeding of uninfected and *T. cruzi*-infected blood. These time scales should help to decipher the early regulation of hemolymph protein expression in response to *T. cruzi* journey in the insect’s digestive tract.

These analyses allowed the identification of (i) the comprehensive hemolymph proteome (ii) the temporal modulation of hemolymph protein expression at 6 h and 24 h post- blood feeding, and (iii) hemolymph protein differential expression in response to *T. cruzi* infection.

### 4.1. R. prolixus Hemolymph Proteome Homeostasis under Blood Feeding Condition

Our analysis led to the identification of 269 proteins (71,54%) in the hemolymph which are constitutively expressed at 6 h and 24 h post-blood feeding (Figure 1 and Table S1). Further bioinformatics analysis of the molecular functions of these proteins revealed that immune, cytoskeleton organization, metabolic pathways, and redox are the most represented processes in the hemolymph proteome independently of the post-feeding time (Figure 2). In addition, this work revealed for the first time the expression at the protein level of numerous *R. prolixus* genes with altered protein expression post-blood ingestion from 6 h to 24 h. Among them, several are of unknown function, which represents 16% of the total hemolymph proteome (Table S1).

As the hemolymph extraction process was achieved by cutting the insect’s legs, some tissue damage of the cuticle and the fat body can lead to wound repair processes [32]. Moreover, we have applied a separation process of hemocytes from plasma by centrifugation which could result in cell lysis leading to hemocytes release of cellular proteins into the plasma. We therefore sorted the identified proteins according to whether they are predicted to be extracellular (hemolymph plasma proteins) or resulting from the alteration of hemocytes and surrounding tissues. Proteins were defined as extracellular if they are secreted through the classic endoplasmic reticulum-Golgi pathway with the guidance of a signal peptide or following UPS pathway. Based on these criteria, the dataset was composed of 241 extracellular proteins representing 64.09% of the total hemolymph proteome and 124 intracellular proteins representing 32.97% of the total proteome (Figure 1B). The remaining eleven proteins (2.92%) were predicted transmembrane proteins (Figure 1B). Previous comparative proteomic analyses of hemocytes and plasma of *Dreissena polymorpha* [33] and *Mytilus edilus* [34] revealed that up to 60% of the plasma proteins could result from hemocytes.

### 4.2. Exploring the Hemolymph Immunoproteins

Hemolymph is the site of important defense mechanisms in insects, relying on the humoral and cellular innate immune response. Insects have developed an array of common strategies to defend themselves against intruders as exemplified by the model *Drosophila* (for review, see [35]); however, recent studies have revealed unique immune adaptations across arthropods taxonomic groups [36]. In this section, a focus will be given to *R. prolixus* hemolymph immune proteins identified in this work, their temporal expression profiles post-blood feeding as well as their modulation by *T. cruzi* infection.

#### 4.2.1. Nonself Perception and Recognition

*R. prolixus* immune response is triggered when its pattern recognition receptors (PRRs) detect pathogen-associated molecular patterns (PAMPs) exposed by pathogens, leading to the activation of effector proteins through signal transduction via the Toll, IMD, and JAK/STAT pathways [3,11]. Hence, PRRs such as PGRPs trigger both Toll and IMD pathway [12,35] and Gram-negative binding proteins (GNBPs) activating the Toll pathway [35,36]. These two major signaling cascades function in a coordinated response between them and other pathways [35,36] to mediate immune response against both Gram-positive and Gram-negative bacteria [37]. Although the activation of these signaling cascades is extracellular, the components of the signaling pathways are intracellular and hence are expected to be absent from the hemolymph plasma. Five PGRPs genes were identified in *R. prolixus* fat body transcriptome encoding thirteen PGRPs isoforms [12]. The authors showed that the isoforms rpPGRP-LC/LAa and rpPGRP-LC/LAb are involved in Gram-negative bacteria recognition and the activation of the IMD pathway [12]. In this work, we have identified a single unique peptide shared by these two isoforms solely in hemolymph at 6 h post-feeding (Table 1). In addition, their expression is triggered 12,8-fold by the parasite ingestion (Table 1). Salcedo-Porras et al. 2021 [12] showed that gene silencing of these two isoforms results into the inhibition of the IMD pathway effectors: defensin B, lysozyme B, and prolixicin at both 8 h and 24 h post-infection. However, it has been proposed that *T. cruzi* is not affected by the AMPs expressed in response to parasite or blood ingestion [38]. Interestingly, the expression pattern of defensins follows that of PGRPs (Table 1). On the other hand, while a single GNBP (RPRC003210) was annotated from *R. prolixus* genome [38,39], we identified three GNBP isoforms (B8LJ39, T1HGN7, and T1I650) in the hemolymph proteome, which expression level is stable at 6 h and 24 h post blood-feeding and post-infection (Table 1). All the identified GNBP isoforms are β-1,3-glucan recognition proteins (GRPs) belonging to the Glycoside Hydrolase Family 16 (GHF16) with altered active site residues in the glucanase domain. B8LJ39 and T1HGN7 are two isoforms of the gene RPRC003210 and belong to the Carbohydrate-Binding Module Family 39 (CBM39). They both have a predicted signal peptide sequence with the absence of a transmembrane domain and a GPI-anchor indicating that they are secreted. On the other hand, T1I650 shares 35% sequence homology with GRP from a moth which was also identified in the fat body of *R. prolixus* in response to thorax injection with a mixture of Gram-positive and Gram-negative bacteria [40]. In addition, T1I650 is the most similar to *Drosophila* GNBP-1 [41] with 38% identity, followed by B8LJ39 with 30%, and T1HGN7 with 29%. However, GNBP-1 was shown to be a GPI-anchored membrane protein [41] while T1I650 sequence, although predicted UPS, it is lacking a signal peptide, a transmembrane domain, and GPI-anchor unless T1I650 N-terminal sequence is incompletely annotated.

In addition to their immune role, these proteins seem to have a digestive implication when they are expressed in the insect’s midgut [42]. Interestingly, we have shown in the comparative proteome of *R. prolixus* midgut [43,44] that T1HU92 (ML domain protein) expression level increases in response to blood feeding. This protein is related to the recognition of pathogen-related molecules [45] and a homologous protein, which expression was also constitutive, was identified in the hemolymph proteome of *A. gambiae* [25]. In the hemolymph, T1HU92 is expressed constitutively from 6 h to 24 h post-feeding.

These published data and our results suggest that different PRRs with different pattern recognition specificities are constitutively present primed to identify different pathogens that may arrive with the blood meal to induce rapidly both humoral and cellular responses. We suggest the adoption of a “watchdog” strategy comparable to the complement alternative pathway [46]. We have also observed a remarkable mounting of a specific pathogen recognition against *T. cruzi* demonstrated by the overexpression of rpPGRP-LC/LAa and rpPGRP-LC/Lab.

#### 4.2.2. Humoral Response through Immune Effectors

Insects use a battery of potent AMPs, such as defensins, lysozymes, attacins, cecropins and prolixicins to combat invading microorganisms through several mechanisms of action, including membrane permeabilization and depolarization [47], targeting lipid II [48], limiting cellular energy, and undermining cell-wall integrity by delocalization of peripheral membrane proteins essential for respiration and cell-wall biosynthesis [49]. AMPs production takes place mainly in the fat body and hemocytes through signal transduction cascades to be released into the hemolymph and the digestive tract [11,50]. Seven classical families of inducible AMPs with several isoforms have been identified in *Drosophila* [51], which act primarily against Gram-negative bacteria and fungi, contributing either additively or synergistically [52]. We have identified seven different AMPs in *R. prolixus* hemolymph in response to blood-feeding or *T. cruzi* infection (Table 1).

**Figure 5:**
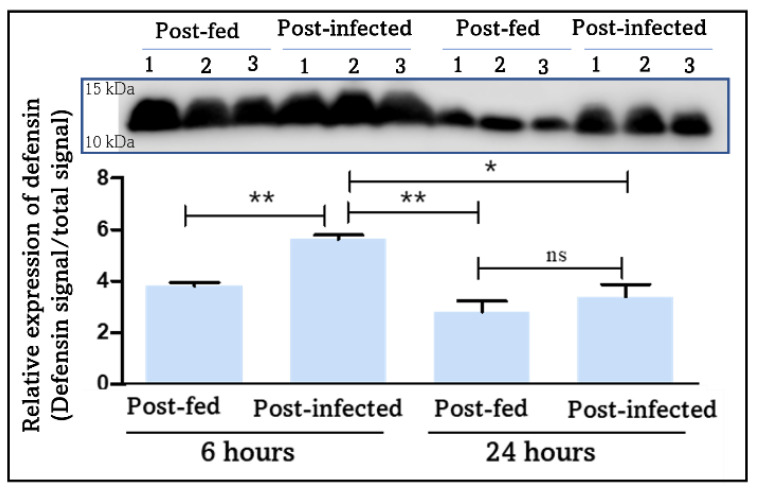
Western blot validation of defensins’ temporal expression profile in the hemolymph at 6 h and 24 h post-blood feeding and *T. cruzi* infection. The relative expression of defensins was calculated by normalizing the band intensity of defensins to the intensity of the total proteins signal. The results are expressed as the mean ± SEM (*n* = 3). Statistical significance is shown by * (* *p* ≤ 0.05 and ** *p* ≤ 0.01), calculated by unpaired *t*-test.

Three defensin isoforms (T1I7B0, R4G8B6, and R4FNJ9) (Table 1) were identified amongst the nine putative defensins encoded by *R. prolixus* genome [39]. They show a seven-fold increase in their expression level 6 h post-*T. cruzi* infection (Table S1). Of note, all three defensins are encoded by the same gene (RPRC012182) and were identified and quantified with a unique and common peptide (WEPAGEITEEHLAR) (Table S1), therefore the three isoforms are indistinguishable, and we cannot affirm which isoforms are unambiguously expressed. Interestingly, this peptide is in the pro-peptide region indicating that we have identified the unprocessed protein which can be activated by a pro-defensin processing enzyme [53]. RPRC012182 clusters separately from hemimetabolous arthropods defensins [39]. Interestingly, these defensins are expressed in the hemolymph 6 h post-blood feeding (Table 1) as it has been observed for defensins A, B, and C transcripts in *R. prolixus* fat body post-hemocoel bacterial injection [54]. However, while defensins A, B, and C transcripts level stays high in *R. prolixus* fat body at 24 h post-hemocoel bacterial injection [54], T1I7B0, R4G8B6, and R4FNJ9 are absent at 24 h (Table 1). However, manual inspection of WEPAGEITEEHLAR peptide revealed its presence also at 24 h, but given its intensity being below the threshold, it was not quantified by MaxQuant algorithm. To confirm the expression of defensins at both 6 h and 24 h and compare their expression level between the different studied conditions, Western blotting experiment using a polyclonal anti-defensin antibody showed a significant increase in the defensins expression level at 6 h post-infection compared to blood-fed condition (*p*-value = 0.0014) (Figure 5). Their expression level in the hemolymph decreases significantly at 24 h post-blood feeding and post-infection (*p*-value = 0.0035 and 0.0118, respectively).

Interestingly, Vieira et al. [55] showed a differential and opposite response of defensins A and C transcripts levels in the fat body 24 h post-oral ingestion of *T. cruzi*. Hence, despite the vectorial life cycle of *T. cruzi* being restricted to the digestive tract, these defensins expression level increases seven-fold at 6 h post-*T. cruzi* infection (Figure 4 and Table 1). Stimulation of the systemic secretion of AMPs in the hemolymph in response to the presence of bacteria or protozoan parasites in the digestive tract has been reported in several insects, including species of *Phlebotomus* [56], *Glossina* [57] and *Drosophila* [58], even without the invasion of the infective pathogens of the hemocoel. Interestingly, a systemic response expression of AMPs in *R. prolixus* hemolymph and fat body following the colonization of its midgut by *T. cruzi* has been reported simultaneously with the local response in the midgut [55]. Moreover, it has been demonstrated that infection of *R. prolixus* with epimastigotes forms of *T. cruzi* Dm28c clone reduces bacteria density, and increases PO and antibacterial activities in the midgut [59]. We can speculate that *T. cruzi* ingestion induces defensin’s expression in the first hours post-infection, which may participate in the control of microbiota population. However, the intensive lysis of *T. cruzi* during the first hours post-feeding leads to the release of a large amount of macromolecules with antigenic properties, which could be responsible of AMPs induction [60].

The other important group of effector proteins with bactericidal activity are lysozymes. Lysozymes are abundant cationic antimicrobial proteins which cleave the accessible peptidoglycan on the cell wall of Gram-positive bacteria releasing fragments that have been related to inflammatory response modulation [61]. *R. prolixus* genome encodes at least seven lysozyme isoforms [38]. In the present work, two isoforms (A9LN32 and T1I5M5) were identified in the hemolymph (Table 1). Their expression is not affected by the presence of *T. cruzi* in the ingested blood at 6 h. However, the expression of T1I5M5 is repressed at 24 h independently of the presence of *T. cruzi,* while A9LN32 expression is steady at 6 h and 24 h. Two isoforms (T1IGM2 and A9LN32) were up-regulated in *R. prolixus* anterior midgut 6 h post-blood feeding [43]. Interestingly, Ursic-Bedoya et al. [62] showed that RpLys-B (A9LN32) aligns with immune related lysozymes from distantly related Lepidoptera or ticks and are expressed principally in the fat body and hemocytes which is confirmed by this work for *R. prolixus.* This is the first time the lysozyme T1I5M5 is identified at the protein level. The protein has 100% sequence identity with lysozyme A0A0P4VPP9, which transcript was identified in *R. neglectus* salivary glands [63]. Interestingly, although this protein was identified in the hemolymph, it misses a signal peptide, unlike the other lysozymes. Alignment analysis with chicken (c-type) and invertebrate-type lysozymes [64] revealed that it belongs to i-type lysozymes.

Three other related glycine-rich AMPs, two diptericin isoforms (D6BJP6 and E6Y430), an attacin (T1I7V7), and a prolixicin (B8QEI8) were identified (Table 1). The expression level of attacin and prolixicin was not affected by thethe parasite in the hemolymph at both studied times suggesting their constitutive expression as it was observed previously for prolixicin transcripts in the fat body and the midgut of *R. prolixus* [65]. However, bacterial and *T. cruzi* injection in the hemolymph resulted in a significant increase in prolixicin transcripts level [65]. Curiously, the authors observed that in vitro application of the recombinant prolixicin was bactericidal, while no significant toxicity was demonstrated against *T. cruzi* [65]. Strikingly, the two diptericin isoforms showed 17,9-fold decrease in their expression level 6 h post-*T. cruzi* infection (Table S1).

Due to their glycine-rich motif, it has been suggested that attacin, diptericin, and prolixicin belong to a monophyletic group with respect to other insect AMPs [66]. Interestingly, we have identified four additional glycine-rich proteins (T1HS54, R4G489, A0A4P6DAB8, and A0A4P6D8R0). Those encoded by RPRC006872 gene, which share 90% sequence identity, show a time-dependent expression profile, and are identified only at 24 h regardless of the physiological condition while T1HS54 is present at 6 h and 24 h (Table 1). RPRC006872 gene products were identified and quantified with five unique and common peptides (Table S1), therefore the three isoforms are indistinguishable, and we cannot affirm which isoforms are unambiguously expressed. 

AMPs have emerged as one of the most powerful actors of the systemic immune response, which is demonstrated by their large diversity and mode of action. Interestingly, this work showed (i) an early, (ii) miscellaneous, and (iii) often constitutive expression of AMPs in the hemolymph regardless of the presence of *T. cruzi* suggesting a rapid, synergetic, and probably additive action of AMPs to efficiently respond to a large range of potential pathogens. Notably, while defensin showed an important induction in the early hours post-*T. cruzi* infection, diptericin showed the opposite pattern suggesting specific induction of defensin by *T. cruzi*.

#### 4.2.3. Melanization and Clotting: Blood-Feeding Induces an Early Immune Response Mediated by PO and Extended by *T. cruzi*

Melanization is an immune response that is triggered by cuticle injury or pathogen invasion of the hemocoel. The activation of this response can be initiated by the recognition of PAMPs by PRRs leading to the synthesis of melanin, resulting in the elimination of the pathogen by melanotic encapsulation or wound healing by sclerotization. PO is the key enzyme in melanin biosynthesis, it mediates through a cascade of reactions the oxidation of tyrosine to quinones which are precursors of melanin that physically encapsulates pathogens [67,68,69]. *T. rangeli* has been shown to be able to inhibit PO activity in *R. prolixus* hemolymph where it establishes before reaching the salivary glands [9,70]. Five POs isoforms (T1I7V8, T1HW62, T1HW22, A0A1B2G385, and A0A1B2G381) were identified in this study (Table 1). Curiously, all isoforms are missing a targeting signal to the secretory pathway. These isoforms are the products of three PPO genes RPRC012380 (T1I7V8 and A0A1B2G385), RPRC008242 (T1HW22), and RPRC008282 (T1HW62 and A0A1B2G381). Isoforms encoded by the same gene are identical besides few amino acids residues additions at the N- or C-termini. These similarities in the PO sequences led us to question the annotation quality of their corresponding CDS. In addition, mass spectrometric data identified pro-enzyme forms, which does not rule out the presence of the active forms. This could be explained by the pre-cleavage of the PPO and the persistence of the cleaved peptides, which have been sequenced by MS/MS.

**Figure 6:**
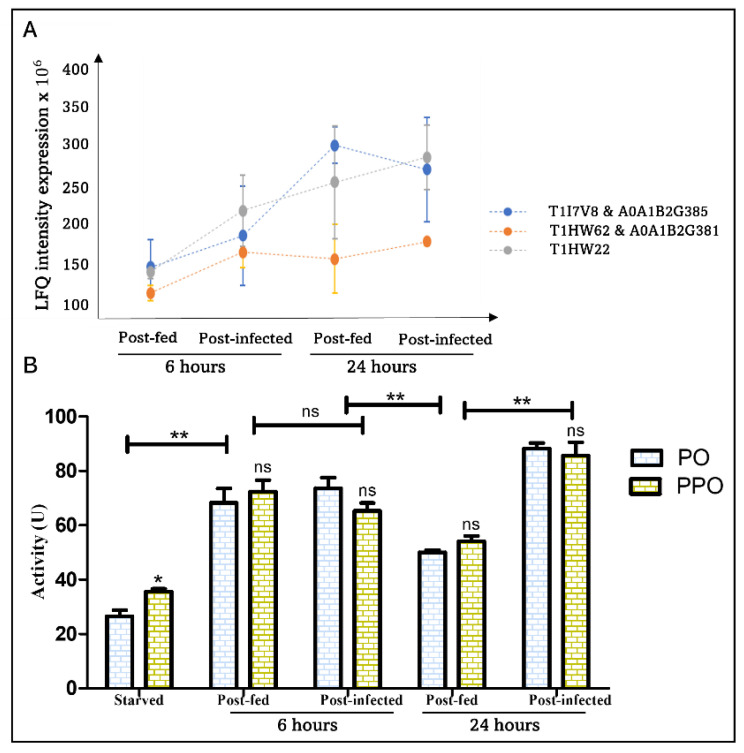
Effect of blood ingestion and *T. cruzi* development on PO and PPO expression and activity in *R. prolixus* hemolymph at 6 h and 24 h post-challenge. (**A**) Profile plot representing the LFQ expression intensity of POs/PPOs isoforms under blood-fed and *T. cruzi* ingestion showing insignificant variation of the protein expression (*n* = 4); (**B**) Evaluation of PO and PPO activity in *R. prolixus* hemolymph from starved, blood-fed and infected insects. The results are expressed as the mean ± SEM (*n* = 3), and statistical significance is shown by * (* *p* ≤ 0.05, ** *p* ≤ 0.01) calculated by unpaired *t*-test.

Quantitative mass spectrometric data did not show significant differential protein expression of PPO/PO in all studied conditions (Table 1 and Table S1). However, the evaluation of PO and PPO activities in the hemolymph at starved, 6 h, and 24 h in blood-fed and *T. cruzi*-infected insects revealed that blood ingestion induces a substantial increase in both PO and PPO activity in the hemolymph independently of the parasite (Figure 6). This remarkable increase in PO activity could be correlated to the observed increase in hemocyte number in response to blood-feeding and consequently the increase in immune components [71] and to the regulation of the gut microbiota after blood ingestion [72]. The PO activity then decreases significantly 24 h after a normal blood meal as observed previously [73], which could be correlated to reduction in the above-mentioned causes but also to PO substrates limitation. Curiously, although PO activity is unaffected by *T. cruzi* at 6 h, it increases significantly at 24 h in the presence of the parasite (Figure 6). It suggests that PO activity, which decreases 24 h post-blood feeding, persists in the presence of *T. cruzi* which might be related to the massive lysis of *T. cruzi*. Since PO and PPO activities are comparable (Figure 6), we can conclude that only the active PO is present in the post-fed conditions.

The active PO is generated by a proteolytic cleavage of PPO zymogen by clip domain serine proteinases (CLIPs), which are specific to invertebrates and act in cascades to modulate several immune responses including coagulation, melanization, and AMPs synthesis through Toll pathway activation [74,75]. The PPO cascade is also tightly controlled by serine protease inhibitors (serpins) to prevent their spontaneous and excessive activation [76].

CLIP proteases are non-digestive serine proteases, apparently unique to invertebrates, and present in the hemolymph. CLIP proteases with a serine protease-like domain with mutated residues of the catalytic triad needed for proteolysis are named serine protease homologs (SPHs). Such CLIP-SPHs with a proteolytic activity deficiency can function as cofactors for PPO activation [77], and contrastingly they can also induce its inhibition [78]. We have identified fifteen SP isoforms of the peptidase family S1 (Table 1). Three isoforms are CLIP-SPs (Table 1) with a conserved serine residue of the protease catalytic triad, a single amino-terminal clip domain, and a secretion signal peptide. They are isoforms of the same gene RPRC003090 which is one of the two annotated CLIP genes from *R. prolixus* genome [38,39]. The three isoforms share more than 97% sequence identity and their identification by MS/MS was based on the same tryptic peptides. Interestingly, the sequenced peptides cover solely the S1 domain probably due to the short and limited number of tryptic peptides of the clip domain. Consequently, we are tempted to suggest that the identified CLIP-SPs are active proteases. The analysis of their temporal expression shows that all isoforms are expressed at 6 h and 24 h post-feeding (Figure 4 and Table 1). Worthy of note, are the three S1 proteins with a catalytically functional triad showing a differential expression upon infection with *T. cruzi*. T1H816 with N-terminal sushi domain has a two-fold expression level increase 6 h post-infection. R4G5A7 and T1I1M7 have a 1,6-fold expression level decrease 6 h post-infection (Table 1 and Table S1).

Once CLIPs are activated, they are tightly regulated by serine proteinase inhibitors (serpins) present in hemolymph plasma [79] to control the toxic by-products of melanization. Proteinases and proteinase inhibitors often exist in pairs. Thus, serine proteinase inhibitors from the serpin family constitute a group of proteins that are likely to be regulators of serine proteinases with a clip domain. Accordingly, four serpins have been identified and their expression profiles are steady at 6 h and 24h post-feeding. However, T1IF83 and R4FJD2 are 1,6-fold down-regulated (Figure 4) by the presence of *T. cruzi* at 6 h post-infection while their expression is unaffected by the parasite at 24 h.

Transglutaminases catalyze the deamidation and transamidation of glutamine, cross-linking of proteins by formation of ε-(γ-glutamyl) lysine isopeptide bonds, which play vital roles in blood clotting, regulation of cellular responses to stress, and formation of the epithelium [3]. It has been shown that transglutaminase activity from both *Drosophila* hemolymph and human blood accumulates on microbial surfaces, leading to their sequestration into the clot. Moreover, *Drosophila* larvae with reduced TG levels show increased mortality after septic injury and susceptibility to a natural infection involving entomopathogenic nematodes and symbiotic bacteria [80]. RNA interference directed against TG reduced the life span of flies reared under conventional conditions and enhanced the expression of AMPs in the IMD pathway [81]. This is probably a consequence of the fact that TG is involved in negative regulation of the IMD pathway. Indeed, TG catalyzes Relish cross-linking suppressing the IMD signaling pathway to enable immune tolerance against resident commensal microbes [81]. We have identified seven TGs among which T1HFV3 and T1HFV2 are only expressed at 6 h post-blood feeding (Table 1). The other isoforms are stably expressed from 6 h to 24 h and might have a role in symbiotic bacteria control [80]. Interestingly, T1HFV3 is drastically down-regulated (20-fold) by *T. cruzi* and could have a different function from the other isoforms. Enthrallingly, Nsango et al. [82] have demonstrated that TG2 from *A. gambiae* has antiparasitic properties contributing to *P. falciparum* killing by the vector [82]. This suggest that TGs might have a putative involvement in the antiparasitic response against *T. cruzi* and be in part responsible of the parasite killing in the anterior midgut and in the hemolymph when artificially injected in the hemocoel [8]. In fact, the two TGs (T1HFR5 and T1HFS7), which showed time-independent expression in the hemolymph (Table 1), were up-regulated in *R. prolixus* midgut in response to blood feeding [43]. Exploring the role of TGs in *R. prolixus* innate immunity could lead to new perspectives on the interaction with the parasite.

#### 4.2.4. Oxidative Stress Related to Immunity

Pathogenic infections in insects leads to the generation of reactive oxygen species (ROS) by the Nox/Duox family of oxidases to promote pathogen killing and elimination. However, if the ROS are not controlled, they can cause damages to biological macromolecules, eventually leading to death. The attenuation of the oxidative response is facilitated by antioxidant enzymes, which play a role of balance between ROS generation and protective function to ensure homeostasis [83]. In this context, we have identified two catalases (T1I0W4 and T1HV37) which expression is not affected by the presence of the parasite, but by the feeding time course (Table 1). Moreover, two SODs (R4FMI6 and T1HRT6) have been identified (Table 1 and Table S1). The quantification of their expression level shows that both isoforms are significantly up-regulated by *T. cruzi* at 6 h post-infection (Figure 4). However, their expression remains stable at 24 h independently of the presence of the parasite. R4FMI6 is predicted to be secreted through the guidance of a signal peptide, while T1HRT6 seems to be intracellular (Table S1). Additionally, a glutathione peroxidase (T1I489) has also been identified to be expressed constitutively in the hemolymph (Table 1).

#### 4.2.5. Pathogen’s Opsonization

Interestingly, we have identified an immunoglobulin I-set domain containing protein (T1HCN4) to be expressed in a constitutive way in all studied conditions (Table 1). Immunoglobulins are proteins involved in several functions, including cell–cell recognition, pathogen opsonization and cell surface receptor. Hemolin is the most studied insect I-set immunoglobulin containing domain, with four domains forming a horseshoe-shaped structure [84]. Hemolin has been identified in the hemolymph of several lepidopteran species [85,86,87]. However, no ortholog has been identified in *D. melanogaster* or *A. gambiae* genomes [88]. It has been shown that hemolin expression is strongly induced by microbial challenge [89,90]. Moreover, hemolin associates with the surface of hemocytes and inhibits their aggregation, suggesting its role in the modulation of hemocyte adhesion during recognition and response to bacterial infections [89]. Hemolin is also able to bind to bacterial surface [91] which confers it a PRR propriety. This is the first time an immunoglobulin I-set domain containing protein has been evidenced in triatomines. The investigation of the immunological proprieties of this protein in triatomines should lead to understanding new immunological processes in these insects.

A putative C1q domain protein (R4FJF3) has also been identified to be expressed in the hemolymph exclusively at 6 h post-feeding. Its expression is highly induced by *T. cruzi* with 7-fold increase compared to the blood feeding condition (Figure 4 and Table S1). Even though a complement-like system has been described in invertebrates [92,93,94], it has been the subject of a very limited number of studies. Interestingly, C1q domain could be involved in the anti-parasitic response. In fact, it has been shown that during the infection of the intermediate host *Biomphalaria glabrata* by the metazoan parasite *Schistosoma mansoni* C1q-like protein expression is up-regulated [95]. The fact that R4FJF3 is modulated by *T. cruzi* suggests its potential anti-parasitic implication. Further investigation is necessary to shed light on the involvement of this protein in the immune response of triatomines and their interaction with *T. cruzi.*

#### 4.2.6. *T. cruzi* Modulates JHBP Expression

We have identified a putative juvenile hormone binding protein (JHBP) (R4FK69) whose expression profile is not affected by feeding time course. However, this protein is up-regulated by the presence of *T. cruzi* at 24 h post-infection (Figure 4 and Table S1). Proteins belonging to this class are crucial for insect development, acting as transporters of JH from the site of its synthesis to different tissues, and protectors of a key insect hormone [96], which is involved in several physiological functions and body homeostasis [97,98]. Little information on the endocrinological regulation of insect immunity by JH exists, but a study in *D. melanogaster* has shown that JH has antagonist effect on the induction of AMPs expression in vitro and in vivo after an immune challenge [99]. Most interestingly, it has recently been shown that JHBP of *A. aegypti* is required for the regulation of innate immune responses and hemocytes development [100]. Indeed, JHBP-deficient insects were characterized by immunosuppression at the humoral and cellular levels, which profoundly affected susceptibility to bacterial infection with a delayed expression of AMPs, severe developmental dysregulation of embryonic and larval hemocytes, and increased differentiation of the granulocyte lineage compared to wild type insects [100]. Therefore, *R. prolixus* JHBP might have an immune implication related to both humoral and cellular response against *T. cruzi* in the hemolymph. The investigation of the function of JHBP in immune competency could provide new insights on triatomines endocrinological regulation of the immune response in triatomines.

## 5. Conclusions

This work allowed the identification of *R. prolixus* hemolymph proteome. The expression pattern of these proteins was followed by quantitative proteomics at 6 h and 24 h after blood-feeding and *T. cruzi* establishment in the insect digestive tract. Our results showed the presence of a broad range of immune proteins in the hemolymph of blood-fed insects which expression is time-dependent. More importantly, we have observed for the first time an induction of a systemic immune response in the hemolymph in response to the early colonization of the vector digestive tract by the protozoan, revealing that the insect is not insensitive to the parasite oral infection. Studies on arthropods defense mechanisms have mainly approached the model organism Drosophila; however, studies on other organisms in particular arthropod vectors are essential to understand the interplay with the pathogens they transmit. Future studies on the proteins highlighted in this work could unravel the mechanisms by which *T. cruzi* is eliminated in the hemolymph and hence might pave the way to new generation strategies to interrupt *T. cruzi* life cycle within the digestive tract and stop its transmission.

## Figures and Tables

**Table 1:** Expression pattern of immunity related proteins identified in *R. prolixus* hemolymph.

Immune Category	IDs	Protein Names	Fold Increase 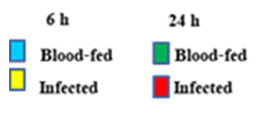
Pattern recognition receptors (PRRs)			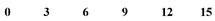
Peptidoglycan recognition receptor (PGRPs)		rpPGRP-LC/LAa	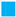
			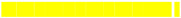
		rpPGRP-LC/LAb	

Gram-negative binding proteins (GNBPs)	B8LJ39	Beta-GRP	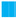
			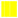
			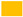
			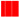
	T1HGN7	GH16 domain-containing protein	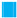
			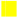
			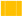
			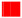
	T1I650	GH16 domain-containing protein	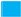
			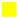
			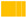
			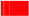
	T1HU92	ML domain-containing protein	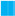
			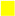
			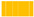
			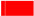
Mucins	T1HEN7	Putative mucin	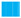
			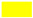
			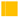
			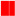
Antimicrobial peptides (AMPs)			
Lysozymes	T1I5M5	Lysozyme	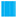
			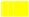
			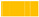
			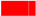
	A9LN32	Lysozyme	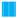
			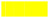


Prolixicins	B8QEI8	Prolixin antimicrobial peptide	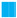
			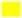
			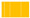
			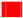
Attacins	T1I7V7	Attacin_C domain-containing protein	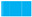
			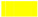
			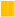
			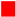
Defensins	T1I7B0	INVERT_DEFENSINS domain-containing protein	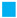
			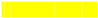


	R4G8B6	Putative defensin	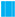
			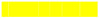


	R4FNJ9	Putative defensin a	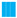
			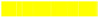


			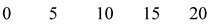
Diptericins	D6BJP6 E6Y430	Diptericin	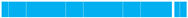
			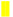
			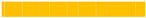
			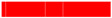
			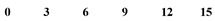
Glycine rich peptides	T1HS54	Uncharacterized protein	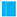
			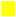
			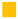
			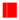
	R4G489	Putative glycine-rich cuticle protein	

			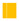
			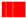
	A0A4P6DAB8	Putative glycine-rich cuticle protein rhodnius neglectus	

			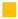
			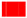
	A0A4P6D8R0	Putative glycine-rich cuticle protein rhodnius neglectus	

			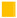
			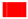
Melanization			
Prophenoloxidases	T1I7V8	Phenoloxidase	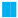
			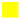
			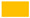
			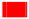
	A0A1B2G385	Phenoloxidase 1 (EC 1.14.18.1)	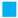
			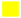
			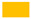
			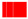
	T1HW62	Phenoloxidase (Fragment)	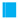
			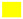
			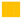
			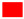
	A0A1B2G381	Phenoloxidase 2 (EC 1.14.18.1)	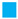
			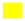
			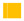
			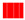
	T1HW22	Phenoloxidase (Fragment)	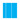
			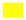
			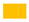
			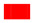
Serine proteases			
Clip-domain SP	T1HGB7	CLIP domain-containing serine protease (EC 3.4.21.-)	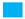
			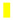
			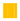
			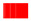
	R4FQA1	CLIP domain-containing serine protease (EC 3.4.21.-)	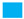
			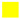
			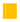
			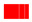
	B8QQQ1	CLIP domain-containing serine protease (EC 3.4.21.-)	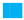
			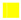
			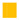
			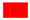
Protease inhibitors			
Serpins	T1IF83	SERPIN domain-containing protein	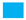
			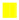
			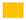
			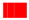
	R4FJD2	Putative serpin length	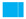
			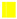
			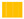
			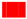
	T1I8D5	SERPIN domain-containing protein	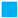
			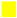
			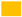
			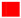
	R4FLP4	Putative serpin length	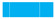
			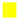
			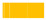
			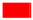
Cystatins	R4FP01	Cystatin (Putative secreted protein)	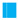
			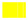
			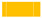
			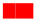
	T1I2F3	Cystatin domain-containing protein	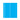
			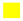
			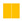
			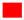
	R4G3U6	Protein with 4 pacifastin inhibitor domains lcmii	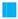
			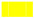
			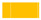
			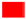
Detoxification			
Superoxide dismutases	R4FMI6	Superoxide dismutase [Cu-Zn] (EC 1.15.1.1)	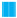
			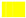
			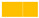
			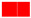
	G1K083	Superoxide dismutase (EC 1.15.1.1) (Fragment)	

			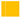
			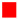
	T1HRT6	Superoxide dismutase [Cu-Zn] (EC 1.15.1.1)	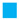
			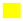
			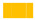
			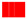
	A0A4P6D9T0	Superoxide dismutase (EC 1.15.1.1) (Fragment)	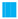
			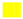
			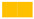
			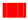
	R4FPK6	Superoxide dismutase (EC 1.15.1.1) (Fragment)	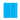
			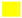
			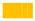
			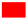
Catalases	T1I0W4	Catalase domain-containing protein (Fragment)	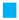
			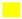


	T1HV37	Catalase domain-containing protein	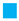
			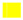


Peroxidases	T1I489	Glutathione peroxidase (Fragment)	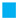
			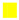
			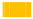
			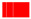
Glutathione S-transferases	T1HVN9	Glutathione S-transferase domain containing protein	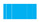
			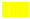
			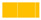
			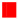
	R4G417	Putative glutathione S-transferase	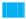
			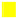
			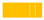
			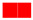
	T1HUM1	Putative glutathione S-transferase	

			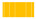
			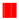
Transglutaminases	T1HFP9	TGc domain-containing protein (Fragment)	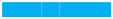
			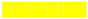
			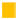
			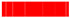
	T1HFS7	TGc domain-containing protein (Fragment)	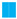
			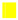
			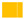
			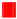
	T1HFV3	TGc domain-containing protein	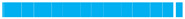
			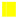


	T1HFR5	TGc domain-containing protein (Fragment)	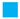
			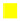
			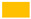
			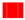
	T1HFR9	TGc domain-containing protein (Fragment)	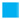
			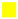
			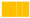
			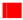
	T1HFV2	Uncharacterized protein	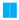
			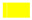


	T1I362	Transglut_C domain-containing protein	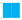
			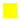
			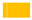
			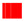
Transferrins	B8LJ43	Transferrin	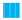
			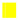
			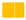
			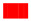
	T1HAU6	Melanotransferrin	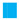
			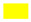
			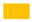
			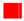
	A0A4P6DAP6	Putative transferrin isoform x4 (Fragment)	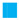
			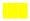
			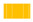
			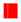
Opsonization	A0A4P6D700	Putative transmembrane protein of the immunoglobulin family of cell adhesion molecules (Fragment)	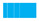
			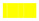
			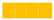
			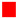
	T1HCN4	I-set domain-containing protein	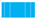
			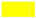
			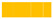
			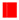
	R4FJF3	Putative c1q domain protein	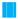
			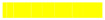



## Data Availability

The mass spectrometry proteomics data have been deposited to the ProteomeXchange Consortium via the PRIDE [101] partner repository with the dataset identifier PXD032787 and 10.6019/PXD032787.

## Supplementary Materials

The following supporting information can be downloaded at: https://www.mdpi.com/article/10.3390/cells11091449/s1, Table S1: *Rhodnius prolixus* hemolymph proteome. Figure S1: Total hemolymph proteins signal.
