# Supplementary material for: Rhodnius prolixus Hemolymph Immuno-Physiology: Deciphering the Systemic Immune Response Triggered by Trypanosoma cruzi Establishment in the Vector Using Quantitative Proteomics
Source: Cells. 2022 Apr 25;11(9):1449. doi: 10.3390/cells11091449 (PMC9104911; doi:10.3390/cells11091449)
Supplement: Supplementary file 1 [file cells-11-01449-s001.zip › Figure S1.pdf]

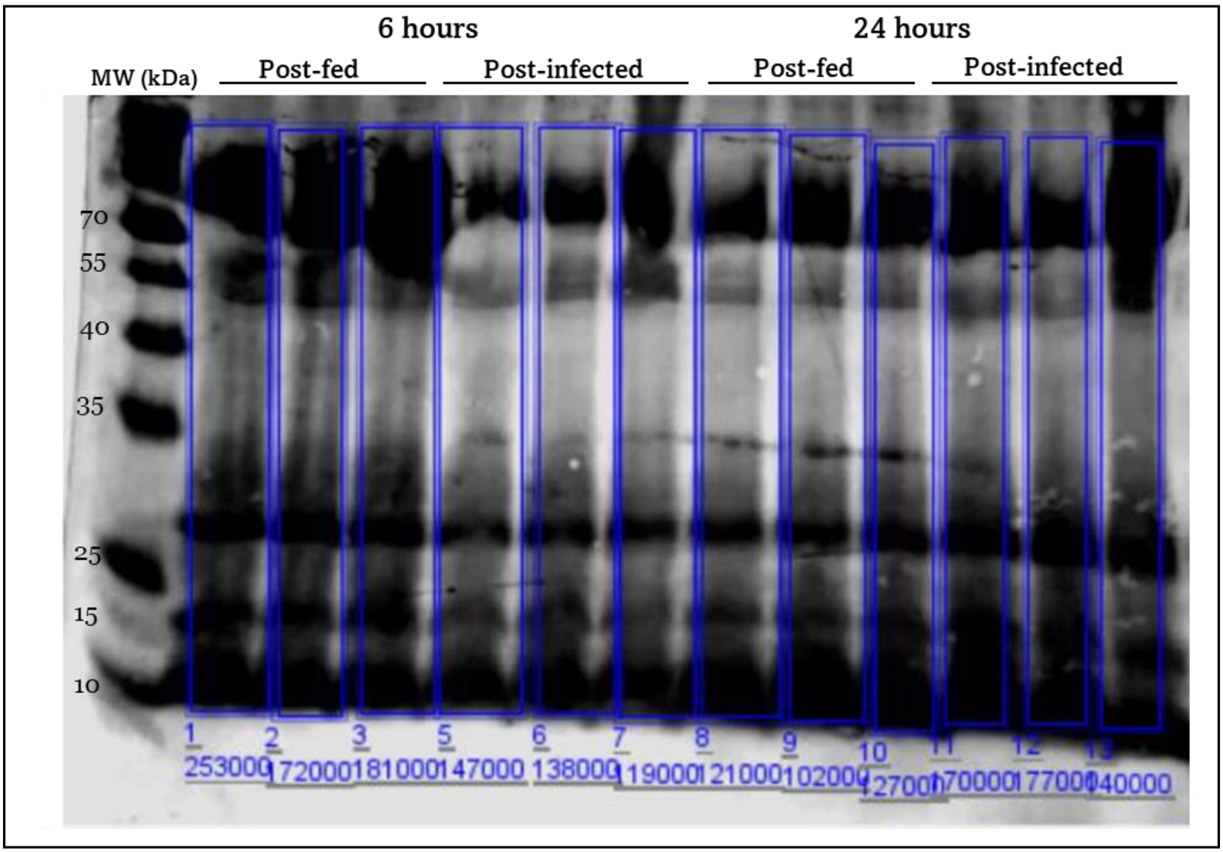

Figure S1 : Total hemolymph proteins signal measure. 15 µg of *Rhodnius prolixus* hemolymph proteins were labelled with cyanine CY5 and separated on 10 % SDS-PAGE gel. After separation, the proteins were transferred into a nitrocellulose membrane and the CY5 labeling was revealed at 700 nm by digital imaging with a CCD camera. In order to compare the different conditions, normalization was based on total proteins. Total protein quantity per lane was measured by summing the signal intensity (pixels) under the defined rectangle (in blue) using LI-COR Image Studio™ acquisition software.
